# Cost-utility analysis of total knee arthroplasty for osteoarthritis in a regional medical center in China

**DOI:** 10.1186/s13561-019-0231-0

**Published:** 2019-05-27

**Authors:** Qi Gui, Xinghuo Zhang, Liang Liu, Feng Zhao, Wenhao Cheng, Yakui Zhang

**Affiliations:** Department of Orthopedics, Beijing Luhe Hospital Affiliated to Capital Medical University, Xinhua South Road #82, Tongzhou, Beijing, 101149 China

**Keywords:** TKA, QALY, Cost analysis, Cost utility ratio, Regional medical center

## Abstract

**Background:**

To analyze the cost-effective ratio of total knee arthroplasty (TKA) in the osteoarthritis (OA) treatment of at a regional medical center in China.

**Methods:**

One hundred thirty-nine patients with osteoarthritis who underwent TKA at the Department of Osteoarthritis in Luhe hospital (Tongzhou, Beijing) from January 2011 to January 2012 were followed up. Their health-related quality of life (HRQoL) was evaluated using Short-Form Health Survey (SF) -36 Chinese version, and compared with those of the normal population to assess quality-adjusted life years (QALYs) gained after surgery for its effectiveness of the treatment. The total expense was the cost of patient hospitalization. The cost per QALY was calculated. The cost-benefit ratio (CBR) was expressed as a ratio of each QALY’s expenditure to per capita gross domestic product (GDP). Factors affecting the cost, including age, gender, length of stay, and ICU experience, were also considered.

**Results:**

The total hospitalization fee was Ұ8,053,736.68, Ұ57,940.55 in average, of which, 81.59% constituted out-of-pocket expenses. The SF-36 scores were as follows: Physical Function: 25.14, Role Physical: 7.12, Bodily Pain: 9.60, Role emotional: 5.58, Vitality: 19.9, Mental Health: 25.84, Social Function: 9.86 and General Health: 21.15. Compared with normal people of the same age and sex, a total of Ұ2487.74 QALYs and Ұ3237.37/QALY were gained, 10% less than the regional GDP per capita. The cost-effective ratio of TKA for osteoarthritis in China was 1: 10.78. The main cost of the patient was the cost of prosthesis (61.78%). The average cost afforded by patients’ themselves was Ұ47,242.64 after the deduction of government subsidies. There were Ұ31,306.64 difference compared with the annual average income of the local people. The cost might be affected by length of stay and ICU experience. Longer stay caused more cost of treatment. Patients who remained in ICU after surgery had higher overall costs and blood transfusion costs.

**Conclusion:**

The factors that affect TKA cost are hospital and postsurgical ICU stay. It is cost-effective for regional medical care center to treat osteoarthritis using TKA economically. However, considering the average income of patients in the area, it is necessary to reduce the cost of the treatment.

**Electronic supplementary material:**

The online version of this article (10.1186/s13561-019-0231-0) contains supplementary material, which is available to authorized users.

## Introduction

With the ageing of the population, the incidence of knee osteoarthritis (OA) in China has risen year by year [1]. According to the Beijing OA study, the prevalence of symptomatic knee OA in the population age > or = 60 years is 15.0% for women and 5.6% for men [1]. The economic burden of knee OA in China is expected to climb further as the aging population increases. Knee replacement surgery is an effective treatment for knee OA to relieve pain, restore function, and improve quality of life (QoL). Unfortunately, medical facilities for total knee arthroplasty (TKA) are not widely available across China, and TKA is primarily performed in metropolis. Although clinically effective, TKA is associated with a high cost, which is an important factor when policy makers determine whether TKA facilities should be provided at regional medical centers [2]. For prefecture-level cities or districts, the economic cost is one of the most important obstacles to effective implementation of TKA. Nowadays, great progress has been made in the construction of prefecture-level regional health care centers. It has become a real problem to be solved in China whether we should provide TKA at regional medical centers.

The Medical Outcomes Survey (MOS) 36-item Short Form Health Survey (SF-36) is a globally recognized QoL assessment tool for evaluation of the quality adjusted life years (QALYs) and it can detect significant changes in the clinical outcomes of OA patients treated with TKA [3]. The Chinese version has been published, and validated in Chinese populations [4, 5]. Cost-utility analysis, which is a tool for evaluating program efficiency [6], translates eligible health utilities into QALYs and can calculate the utilities in an integrated manner [7]. In this method, the cost was calculated using Kopeck, and utility was counted by QALYs. The evaluation of the program was the acquisition of the cost benefit ratio [8].

In China, studies on TKA for knee OA have focused on its technical feasibility and long-term survival of patients [9, 10]. Although the cost-effectiveness of TKA has been assessed in numerous high-quality studies on other populations [6], such data is scant for Chinese patients. Waimann reported a mean total incremental cost and a mean total cost per TKA of $20,133 and $24,435, respectively, of which 65% was attributable to total TKA-related costs and 25% to indirect costs [11]. They found that TKA was cost-effective for end-stage knee OA [11]. Similarly, Losina et al. found that TKA was highly cost-effective, even with an incremental cost of $18,300 per QALY gained, with hospitalization being the major factor governing the costs [12]. The authors also observed that cost-effectiveness varied considerably depending upon the capacity of the medical centers and level of risk associated with the patients [12]. Given the differences in health policies between different countries, the results of cost-effectiveness analysis from other countries may not be applicable to China. As the prefecture-level region is underdeveloped and TKA-associated costs are substantially high, the assessment of cost-utility analysis and “value for money invested” is necessary before expanding TKA facilities to regional medical centers in China. Therefore, our study aimed to evaluate cost benefit ratio and carry out cost-utility analysis at a regional medical center in Beijing to provide insight into the health economic status of the prefecture-level region of China.

## Patients and methods

### Patients

The prospective study was approved by the Hospital Ethics Committee and all patients provided written informed consent.

The study enrolled patients who underwent TKA between January 2011 and January 2012 at Luhe Hospital (Tongzhou, Beijing). Follow-up exclusion criteria were: 1) patients underwent TKA due to other reasons; 2) patients who suffered from OA did not receive TKA; 3) patients underwent bilateral TKA in different hospitals; 4) patients died from other kinds of diseases; 5) patients did not voluntarily conduct questionnaires; 6) patients underwent other operations after TKA.

## Methods

### Questionnaires

A semi-structured questionnaire was used to collect baseline data such as age, gender, and surgical details (TKA and postsurgical complications).

SF-36 Chinese version: The self-administered questionnaire assesses health-related QoL across 8 domains of functional health and well-being as follows: Physical Function (PF), Role Physical (RP), Bodily Pain (BP), General Health (GH), Vitality (VT), Social Function (SF), Role emotional (RE), Mental Health (MH), and Report Health Transition (HT). As the Report Health Transition domain is related to the tendency of health change, it was not included in our research [3, 13]. SF-36 values for Chinese general population from a previous study were used as control (henceforth referred as “Chinese norm”) (Additional file 1: Table S1) [14].

### Follow-up

Patients were followed up via telephone calls or home visit if telephone follow-up was not available. Patients were assessed by SF-36 at 3, 6 and 12 months post surgery and every 3 months thereafter.

### Scoring method using SF-36

**Fig. 1:**
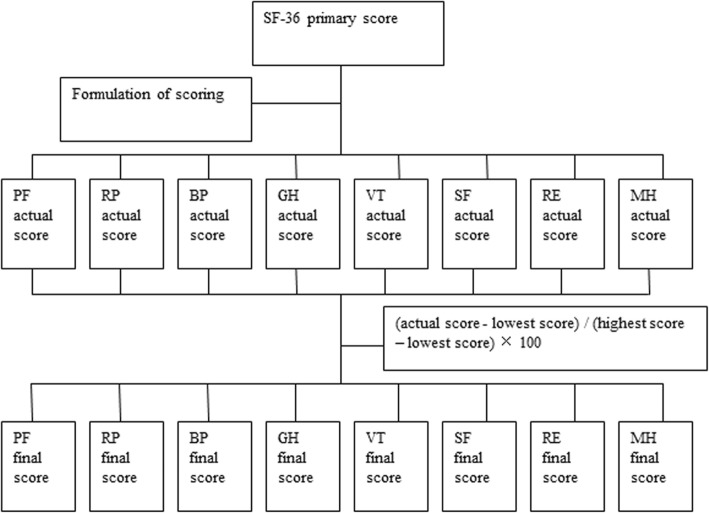
The procedure of each aspect’s final score

Scores on the 8 health-related scales of SF-36 were calculated using standard methods (Fig. 1 and Additional file 2: Table S2). To obtain the final score, the following formula was used:

$$ \mathrm{Final}\ \mathrm{score}=\left(\frac{\mathrm{actual}\ \mathrm{score}-\mathrm{theoretical}\ \mathrm{lowest}\ \mathrm{score}}{\mathrm{theoretical}\ \mathrm{highest}\ \mathrm{score}-\mathrm{theoretical}\ \mathrm{lowest}\ \mathrm{score}}\right)\times 100. $$

The final scores thus obtained were compared against the Chinese norm and converted into standard Z scores:

$$ \mathrm{Z}=\frac{\mathrm{mean}\ \mathrm{score}\ \mathrm{of}\ \mathrm{patients}-\mathrm{mean}\ \mathrm{score}\ \mathrm{of}\ \mathrm{Chinese} \operatorname {norm}}{\mathrm{standard}\ \mathrm{deviation}\ \mathrm{of}\ \mathrm{Chinese} \operatorname {norm}}. $$

### Cost calculation

As the main expenditure for TKA occurs during the inpatient period, the TKA-related cost for each patient during this period was assumed as the total cost of the procedure.

### Effect calculation

**Fig. 2:**
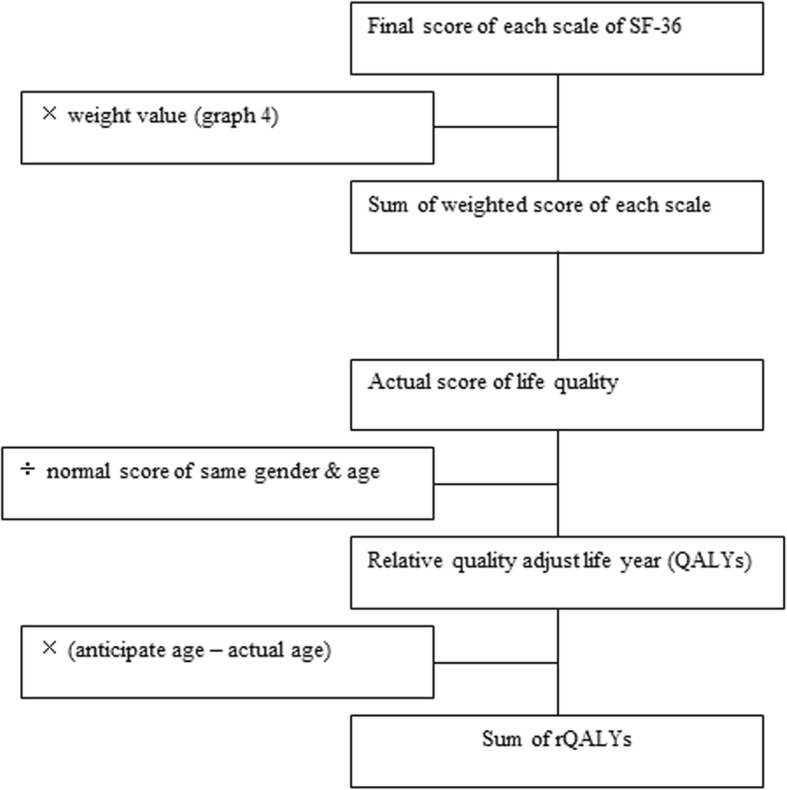
The flowchart for obtaining the sum of rQALYs

Effect was calculated using the QALY scores. We used a weighted average of the Chinese norm as the baseline value (1 QALY). By comparing the weighted average of patients against that of the Chinese norm for the same age and gender, the actual QALY (rQALY) were calculated. Assuming a mean life expectancy of 80.18 years in Beijing [15], the expected QALYs were calculated using the following formula: QALYs = (life expectancy-present age) × rQALY. The total effect was calculated as the sum of all QALYs (Figs. 1 and 2).

### Factors affecting cost

The effects of age, gender, length of hospital stay, and postsurgical ICU stay on the cost of TKA were analyzed, separately.

### Cost utility ratio (CUR)

CUR = total cost / total effect (Yuan / QALY, 1 USD = 6.20 RMB as of April 30, 2015)

### Cost-benefit ratio

Because the patients lived in a defined geographical area, the GDP of that local region was used to calculate the monetary value of each QALY. The total treatment cost divided by the monetary value of QALYs was considered the cost benefit ratio (CBR):

$$ \mathrm{CBR}=\frac{\mathrm{total}\ \mathrm{cost}}{\left(\mathrm{GDP}\ \mathrm{per}\ \mathrm{capita}\times \mathrm{total}\ \mathrm{QALYs}\right)}. $$

### Statistical methods

Cost utility ratio and cost benefit ratio analyses were conducted. The cost of surgery according to individual patient’s characteristics such as gender, age, length of hospital stays, and postsurgical ICU stay were compared using one-way ANOVA. All *P* values are 2-sided, with a significance level of 0.05. All statistical analyses were conducted using the Statistical Package for Social Sciences (SPSS) 17.0 for Windows (SPSS Inc., Chicago, IL).

## Results

### Demographic and baseline characteristics of the study population

One hundred fifty-nine patients were assessed for eligibility. Eight patients who underwent TKA due to rheumatoid arthritis were excluded. Furthermore, three patients who had TKA due to post-traumatic arthritis, four patients who had bilateral TKA, and one patient who underwent open reduction and internal fixation of the same-side ankle joint surgery after TKA were excluded. In addition, two patients who were not residents of Tongzhou and two who were lost to follow up were also not included. Eventually, 139 patients had complete data and the follow-up length was 15.2 ± 3.0 months. Finally, 139 patients (64.32 ± 7.55 years old; 81.29% women) were included in the study. The mean follow-up duration was 15.2 ± 2.9 months. Overall, 25 (18.0%) patients underwent bilateral TKA: 15 (21.1%) patients were aged < 65 years and 10 (14.7%) aged ≥65 years (*P* = 0.32). The mean age of patients who underwent unilateral TKA is 64.67 ± 7.89 years old). There were no major postsurgical complications. The scores of the SF-36 are as follows: PF: 25.14, RP: 7.12, RE: 5.58, BP: 9.60, VT: 19.9, MH: 25.84, SF: 9.86 and GH: 21.15. The patient scores were compared with the Chinese norm for the same gender and age to calculate the rQALY. According to the report of the Chinese’s Department of Health [15], the assumed mean life expectancy of Beijing residents was 80.18 years in 2010, the expected QALY was calculated as QALY = (80.18- actual age) × rQALY (normal person’s weight score is shown in Additional file 3: Table S3). The total QALYs were 2487.74, and the average QALY gain per person is 17.90 ± 9.52.

**Fig. 3:**
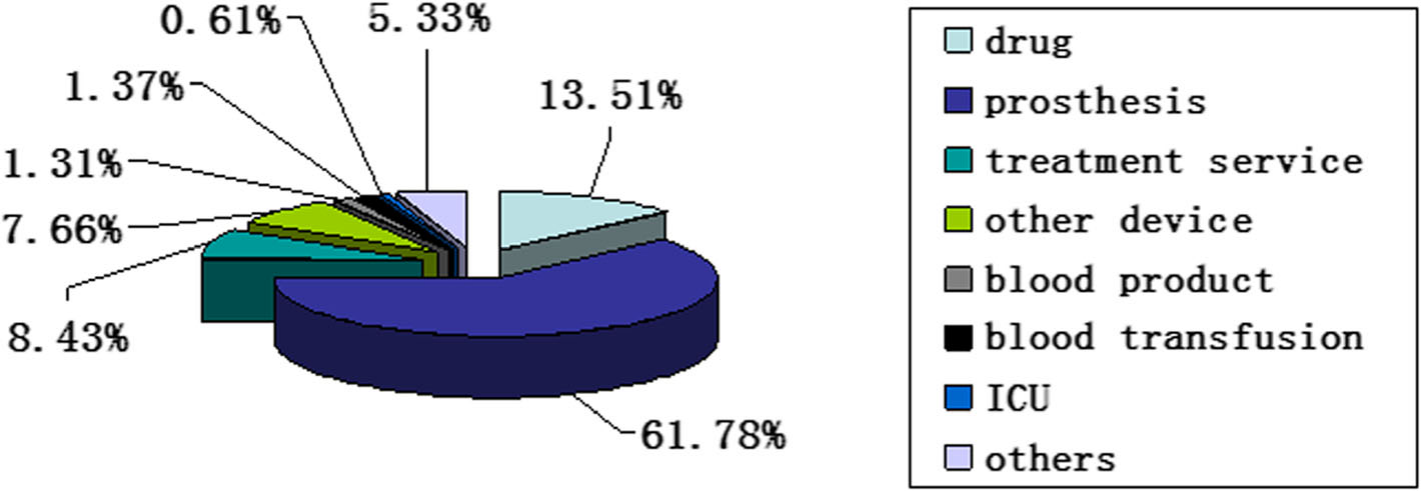
The cost composition of the TKA treatment at Tongzhou Regional Medical Center of Beijing, China

Because no cost was attributed to the complications of TKA, only the cost during hospitalization for each patient was considered. The total cost of all TKAs was RMB Ұ8,053,736.68 (~£89,000 or ~$1,232,000). Therefore, the cost of each QALY gained was RMB Ұ3237.37 (~£350 or ~$500). The mean cost per patient was RMB Ұ57,940.55 (~£6300 or ~$8900), whereas the average out-of-pocket expense for each patient was RMB Ұ47,272.30 (~£5200 or ~$7200). The cost benefit ratio was 1:10.78, calculated as the ratio of the cost per QALY to the GDP per capita (RMB Ұ34,897.40 [~£3800 or $5300] in 2012) [10]. The cost of prosthesis, drugs, and other applications was 61.78%, 13.51%, and 7.66%, respectively, of the total cost. The cost of treatment services (surgery and anesthesia) was only 8.43% (Fig. 3).

A comparison of TKA costs based on factors such as gender, age, length of hospital stays, and postsurgical ICU stay (Table 1) showed that length of hospital stay and postsurgical ICU stay were significantly associated with total TKA cost. Longer hospital stays were associated with higher TKA costs. Patients with postsurgical ICU stay incurred significantly higher total costs and costs of blood transfusion. Despite no significant difference was observed in cost by age groups, younger patients incurred higher costs. Although gender was not related to cost, men incurred lower costs of blood transfusion; however, this difference was not statistically significant.

**Table 1:** Expenses by categories for TKA

Variables	No.	Total	Drugs	Prosthesis	Treatment^a^	Devices^b^	Blood	Cost/QALY
Age, years								
≥65	68	56,269.46	7868.99	33,878.10	4908.32	4413.55	808.18	2752.37
<65	71	59,541.03	7785.64	37,631.65	4865.65	4461.26	714.31	5960.78
Gender								
Male	26	51,712.27	7443.76	31,631.35	4308.19	3952.92	499.31	7083.72
Female	113	59,373.61	7914.46	36,753.48	5019.59	4549.51	820.27	9403.7
Length of stay, days								
10–19	60	52,198.43*	6651.03*	33,050.40	4283.33*	4023.32*	410.53*	3559.88
20–29	56	58,053.63*	7559.36*	36,569.87	4598.00*	4364.01*	974.54*	4626.65
≥30	23	72,644.66*	11,542.88*	41,070.48	7162.60*	5699.42*	1150.70*	5568.10
ICU stay								
Yes	17	68,099.53*	8815.39	40,861.43	5788.48	5200.10	1241.06*	4110.24
No	122	56,524.96*	7688.61	35,089.46	4760.84	4331.71	693.23*	5841.29

^a^Cost of operations; ^b^costs for use of devices during hospital stay; * *P* < 0.05, one-way ANOVA test

Cost Benefit ratio: the cost of each QALY acquainted was calculated as follows: “total cost ÷ total utilities”, and the outcome was RMB Ұ3237.37/QALY. The actual value of each QALY was represented with the GDP of the local region in 2012. That was RMB Ұ34,897.40 (total RMB Ұ45,052.540 thousand, resident population 1291 thousand [10]). The cost benefit ratio of the TKA in regional medical center was 1: 10.78. It suggests that we could get RMB Ұ10.78 in benefit for the whole society if we invest RMB Ұ1 in TKA treatment for OA.

As all patients were divided into five groups based on age (≤60, 61–65, 66–70,71–75, > 75), the benefit of QALY was shown in the Table 2. The average QALY and the CBR of younger patients are higher than that of older patients, while the cost of a single QALY is lower than that of older patients.

**Table 2:** Age based benefits of QALY of the TKA treatment at Tongzhou Regional Medical Center of Beijing, China

Age	Average QALY (Year)	Cost/QALY(¥, RMB)	Cost-benefit Ratio
≤60	27.31	2214.58	1: 15.76
61–65	19.49	3051.03	1: 11.44
66–70	14.31	4024.48	1: 8.67
71–75	7.7	7501.7	1: 4.65
> 75	2.23	20,019.96	1: 1.74

## Discussion

Although previous studies have reported the cost-effectiveness of TKA in knee OA [11, 12, 16, 17], data from China are unavailable. Our study evaluated cost benefit ratio and conducted cost-utility analysis to better understand the health economics of providing facilities for TKA for knee OA at regional medical care centers in China. Since Luhe Hospital (Tong Zhou, Beijing) is in a region whose average per capita income (RMB Ұ34,897.40) is close to China’s GDP (RMB Ұ39,500), this hospital is a representative of other regional hospitals in China [18, 19]. In addition, as hospitals in China are nationalized, health care costs are comparable across the country. Our results show that this intervention is highly cost-effective, and factors such as length of hospital stay and postsurgical ICU stay were significantly associated with higher total costs of TKA.

Western models suggest a threshold of healthcare expenditure in the “range of acceptable cost-effectiveness” from £20,000 to £30,000 per QALY or slightly higher [20]. This study showed a cost of RMB Ұ3237.37 (~£350 or ~$500) per QALY gained, which is much lower than cost reported in Western countries, suggesting that TKA is highly cost-effective. Of note, these costs are not country specific. The “Choosing Interventions that are Cost-Effective” (CHOICE) project, a WHO initiative that provides guidelines to assess the cost-effectiveness of an intervention, is being used to determine country-specific thresholds. Based on the cost per QALY gained in contrast to the GDP of the country, interventions are classified as highly cost-effective (below the GDP per capita), cost-effective (1–3 times the GDP per capita), and not cost-effective (over 3 times the GDP per capita), per the WHO-CHOICE guidelines [21]. Our cost-utility analysis showed that the cost of TKA performed at the regional medical center was RMB Ұ3237.37/QALY gained. The per capita GDP for the study area was RMB Ұ34,897.40 in 2012. Therefore, < 10% of per capita GDP is needed to gain 1 QALY by using TKA, implying that TKA is highly cost-effective. The cost benefit ratio of TKA (1:10.78) in the regional medical center indicates that RMB Ұ1 investment in TKA for knee OA would yield a RMB Ұ10.78 benefit for the society. However, the budget of medical care in China is only 1.39% of the GDP. As > 80% of TKA expenses were out-of-pocket, with a mean out-of-pocket expenditure of RMB Ұ47,272.30 (~$7000) for patients, which is almost thrice the 2012 mean per capita income of RMB Ұ15,936 of the study area [18, 19], this cost may be prohibitively high from an individual’s perspective. In addition, the average expense afforded by the society is RMB Ұ485.07 (1.39% of GDP), which is well below the expenditure required for TKA. Interestingly, a survey in patients with total hip and total knee arthroplasty from multiple centers in the US revealed that patients were willing to pay a considerable amount of money out-of-pocket ($12,797 for TKA) accounting to nearly 50% of the total costs for these highly successful procedures [12] Although this perspective may not be applicable to the setting in China, it is noteworthy that patients are valuing and willing to spend a lot of money out-of-pocket to receive TKA.

According to our analysis, the cost of prosthesis accounts for 61.78% of the total cost. The costs of drugs and other applications were 13.51% and 7.66%, respectively. The treatment cost (surgery and anesthesia) was only 8.43%. These findings are contrary to studies in other countries. In a US study, the surgical fee was 20.21% and the cost of prosthesis for primary TKA was 36.93% (although it was one of the most expensive prostheses available) [13], whereas a Finnish study reported a TKA prosthesis cost of 24% [22]. The lower costs of surgery and anesthesia in our study may be attributed to universal healthcare access and government subsidies in China. The Chinese government supervises the medical industry. Most of surgeons/anesthetists in public institutions receive salaries from the government; hence, costs of surgery and other consumables are relatively low. Thus, reducing the high cost of imported prostheses may help reduce China’s TKA costs. This highlights the urgent need to study the development and local production of these artificial limbs in China.

Various factors could affect the cost of TKA. We evaluated the effects of gender, age, length of hospital stays, and postsurgical ICU stay during the inpatient period. Longer hospital and postsurgical ICU stays were associated with higher TKA costs due to higher cost of consumables, drugs, and treatment. Contrary to expectations, the cost was higher for younger versus older patients (RMB Ұ59,541.03 vs. RMB Ұ56,269.46). A probable reason may be the higher proportion of bilateral TKA in younger patients (21.1%) compared with older patients (14.7%) as bilateral TKA is costlier than unilateral TKA. Interestingly, no association was observed between gender and higher TKA costs. Longer hospital stay, and postsurgical ICU stay were significantly associated with higher total costs and costs for blood transfusion.

The Global Burden of Disease study 2010 found that hip and knee OA together was ranked as the 11th contributor to global disability and the 38th in DALYs; the global age-standardized prevalence of knee OA was 3.8% (95% CI, 3.6%–4.1%) [23]. The prevalence of knee OA in China is high, which is expected to increase further with population aging. Related pain and disability affect the QoL and may also affect the productivity of affected individuals. The cost of medicines, the drop-in productivity and the needs of nursing staff may result in large costs for individuals and society. Our study illustrated the cost-effectiveness of TKA performed at regional centers. The high prevalence and relative unavailability of knee replacements in China require additional medical services, which may be achieved by providing TKA facilities at regional-level hospitals. Given the recent evidence from the small hospital, some other factors should also be considered. Katz et al. in a US-based study reported that patients undergoing primary TKAs in low-volume hospitals, performed by low-capacity surgeons, had worse functional outcomes 2 years after TKA [24]. Thus, in addition to cost-utility analysis, future studies must assess long-term surgical outcomes in terms of pain, knee mobility, and patient satisfaction in regional medical centers.

According to our analysis, TKA is highly cost-effective, with < 10% GDP per capita expenditure per QALY gained. However, this study also has some limitations. To estimate QALYs gained after surgery, the pre-surgical QoL was assumed to be zero. For cost calculation, only the inpatient period costs were included. However, the results would not change considerably even after adding costs incurred after the inpatient period. Our analyses were based on data from a single regional medical care center and did not involve economic modeling. The results may vary across different scenarios, which must be considered in economic decision making [25]. This study helps to show that regional medical centers have required resources to successfully perform TKA procedures. We believe that our findings are valuable in guiding Chinese TKA’s medical policy decision-making, especially in extending facilities to regional medical centers because TKA is highly cost-effective in addition to be a reliable and popular surgical procedure.

As we known, the actual total cost of patients younger than 65 years old is higher than that of patients over 65 years old, in part because the patients who choose bilateral TKA surgery are more than the older group. If the total income and cost of QALY in the younger age group were taken into consideration, the average QALY and the CBR of younger patients are higher than that of older patients, while the cost of a single QALY is lower than that of older patients, suggesting the younger patients can yield the better benefits. So, for younger patients who needs the bilateral TKA surgery, the coverage of public health insurance should be appropriately increased.

The patients aged > 75 years expensed significantly higher than other groups, but their personal QALY income significantly lower than other groups, suggesting that older patients need to consume more resources if they achieve the same quality of life. Therefore, it is necessary to increase the medical insurance coverage for senior patients to reduce the economic pressure of individuals. In addition, surgical treatment has a lower overall benefit for quality of life improvement for older patients due to the limited number of quality-adjusted life years. At the same time, the probability and prevalence of preoperative complications in elderly patients are higher. Therefore, from the aspect of health policy, it is worth more researches whether the strict admission for TKA of older patients (age > 75) should be adopted to improve the economic utility.At present, China’s public health insurance policy has low coverage in TKA and does not consider the impact of age on the health care economy. From the perspective CBR, the average economic burden is heavy (TKA averages ¥47,272.30 vs annual average income ¥15,936). In the sum, it is necessary to increase the public investment for TKA surgery at the level of public health policy.

## Conclusion

The present study showed that TKA performed at a regional medical center for knee OA was highly cost-effective: < 10% of per capita GDP is required to gain 1 QALY. However, the TKA cost was higher than the average income of the local population. The cost of prosthesis constituted a major expense for TKA. Length of hospital stays, and postsurgical ICU stay considerably increased TKA cost.

## Additional files

Additional file 1: **Table S1.** SF-36 score and weight score of normal Chinese people. (DOCX 13 kb)

Additional file 2: **Table S2.** The scoring formulation and range of SF-36. (DOCX 12 kb)

Additional file 3: **Table S3.** SF-36 weights of 8 domain scales. (DOCX 12 kb)

## Abbreviations

BP: Bodily Pain
CBR: Cost-benefit ratio
GDP: Gross domestic product
GH: General Health
HT: Report Health Transition
MH: Mental Health
MOS: Medical Outcomes Survey
OA: Osteoarthritis
PF: Physical Function
QALYs: Quality-adjusted life years
QoL: Quality of life
RE: Role emotional
RP: Role Physical
SF: Short-Form Health Survey
SF: Social Function
TKA: Total knee arthroplasty
VT: Vitality

## References

[1] Wei Y, Ling X, Qin M, et al. Epidemiology of the prevalence of elderly knee osteoarthritis in Beijing: comparison with that of whites in the United States. Chin J Radiol. 2005;39(1):67–71.

[2] Peersman Geert, Jak Wouter, Vandenlangenbergh Tom, Jans Christophe, Cartier Philippe, Fennema Peter (2014). Cost-effectiveness of unicondylar versus total knee arthroplasty: a Markov model analysis. The Knee.

[3] Gandek B, Ware JE, Aaronson NK (1998). Tests of data quality, scaling assumptions, and reliability of the SF-36 in eleven countries: results from the IQOLA Project. International Quality of Life Assessment. J Clin Epidemiol.

[4] Li L, Wang HM, Shen Y (2003). Chinese SF-36 Health Survey: translation, cultural adaptation, validation, and normalisation. J Epidemiol Community Health.

[5] Hu J, Gruber KJ, Hsueh KH (2010). Psychometric properties of the Chinese version of the SF-36 in older adults with diabetes in Beijing, China. Diabetes Res Clin Pract.

[6] Daigle ME, Weinstein AM, Katz JN (2012). The cost-effectiveness of total joint arthroplasty: a systematic review of published literature. Best Pract Res Clin Rheumatol.

[7] Konopka JF, Lee YY, Su EP, McLawhorn AS. Quality-Adjusted Life Years After Hip and Knee Arthroplasty Health-Related Quality of Life After 12,782 Joint Replacements JB JS Open Access. 2018;3(3):e0007 10.2106/JBJS.OA.18.00007 PMC6242318 30533590

[8] Krummenauer F, Wolf C, Günther KP (2009). Clinical benefit and cost effectiveness of total knee arthroplasty in the older patient. Eur J Med Res.

[9] Yue B, Wang J, Wang Y (2014). How the gender or morphological specific TKA prosthesis improves the component fit in the Chinese population?. J Arthroplast.

[10] Feng B, Weng X, Lin J (2013). Long-term follow-up of cemented fixed-bearing total knee arthroplasty in a Chinese population: A survival analysis of more than 10 years. J Arthroplast.

[11] Waimann CA, Fernandez-Mazarambroz RJ, Cantor SB (2014). Cost-effectivenessof total knee replacement: a prospectivecohort study. Arthritis Care Res (Hoboken).

[12] Losina E, Walensky RP, Kessler CL (2009). Cost-effectiveness of total knee arthroplasty in the United States: patient risk and hospital volume. Arch Intern Med.

[13] Courtney PM, Howard M, Goyal N, Schwarzkopf R, Schnaser E, Sheth NP (2015). How much do patients value total hip and knee arthroplasty? A prospective, multicenter study. J Arthroplast.

[14] Li NX, Liu CJ, Li J, Ren XH (2001). The norms of SF-36 dimension scores in urban and rural residents of Sichuan province (in Chinese). Hua Xi Yi Ke Da Xue Xue Bao.

[15] Annals demographic data of Beijing in 2010, National Bureau of Statistics of the people’s republic of China, available from: http://data.stats.gov.cn/search/

[16] Slover J, Hoffman MV, Malchau H (2009). A cost-effectiveness analysis of the arthroplasty options for displaced femoral neck fractures in the active, healthy, elderly population. J Arthroplast.

[17] Li CS, Seeger T, Auhuber TC (2013). Cost-effectiveness and economic impact of the KineSpring knee implant system in the treatment for knee osteoarthritis. Knee Surg Sports Traumatol Arthrosc.

[18] Summarize of economical data of Tongzhou, Beijing, China, from 2005–2012, available from: http://www.beijing.gov.cn/zfxxgk/tzq11L024/tjxx53/2013-02/04/content_40774.shtml

[19] Annals economic data of China in 2012, National Bureau of Statistics of the people’s republic of China, available from: http://data.stats.gov.cn/easyquery.htm?cn=E0103&zb=A0303&reg=110000&sj=2010

[20] Devlin N, Parkin D (2004). Does NICE have a cost-effectiveness threshold and what other factors influence its decisions? A binary choice analysis. Health Econ.

[21] WHO (2014). Cost effectiveness and strategic planning (WHO-CHOICE): Cost-effectiveness thresholds.

[22] Lavernia CJ, Drakeford MK, Tsao AK, Gittelsohn A, Krackow KA, Hungerford DS (1995). Revision and primary hip and knee arthroplasty: a cost analysis. ClinOrthopRelat Res.

[23] Cross M, Smith E, Hoy D (2014). The global burden of hip and knee osteoarthritis: estimates from the Global Burden of Disease 2010 study. Ann Rheum Dis.

[24] Katz JN, Mahomed NN, Baron JA (2007). Association of hospital and surgeon procedure volume with patient-centered outcomes of total knee replacement in a population-based cohort of patients age 65 years and older. Arthritis Rheum.

[25] Krummenauer F, Guenther KP, Kirschner S (2011). Cost effectiveness of total knee arthroplasty froma health care providers’ perspective before and after introduction of an interdisciplinary clinical pathway - is investment always improvement?. BMC Health Serv Res.

