# Supplementary material for: Cost-utility analysis of total knee arthroplasty for osteoarthritis in a regional medical center in China
Source: Health Econ Rev. 2019 May 27;9:15. doi: 10.1186/s13561-019-0231-0 (PMC6734290; doi:10.1186/s13561-019-0231-0)
Supplement: Supplementary file 1 — Table S1. SF-36 score and weight score of normal Chinese people. (DOCX 13 kb) [file 13561_2019_231_MOESM1_ESM.docx]

**Supplementary Table 1**: SF-36 score and weight score of normal Chinese people

| Age, years | Gender | PF | RP | BP | GH | RE | VT | MH | SF | Weight score |
| --- | --- | --- | --- | --- | --- | --- | --- | --- | --- | --- |
| 16 - 39 | M | 91.30 | 81.00 | 88.18 | 70.45 | 65.78 | 65.41 | 65.06 | 78.51 | 73.98 |
|  | F | 87.69 | 79.55 | 82.45 | 67.98 | 65.94 | 63.71 | 64.65 | 78.76 | 72.30 |
| 40 - 59 | M | 86.09 | 84.66 | 85.53 | 61.92 | 83.33 | 64.91 | 69.99 | 81.35 | 75.90 |
|  | F | 81.89 | 81.03 | 76.98 | 64.63 | 83.33 | 68.92 | 72.54 | 79.15 | 75.52 |
| ≥ 60 | M | 83.74 | 82.35 | 82.53 | 67.75 | 83.33 | 75.51 | 78.83 | 83.31 | 79.27 |
|  | F | 79.13 | 59.00 | 69.42 | 62.84 | 77.33 | 66.67 | 70.78 | 78.45 | 70.27 |

BP: Bodily Pain; GH: General Health; MH: Mental Health; PF: Physical Function; RE: Role emotional; RP: Role Physical; SF: Social Function; VT: Vitality
