# Supplementary material for: Cost-utility analysis of total knee arthroplasty for osteoarthritis in a regional medical center in China
Source: Health Econ Rev. 2019 May 27;9:15. doi: 10.1186/s13561-019-0231-0 (PMC6734290; doi:10.1186/s13561-019-0231-0)
Supplement: Supplementary file 2 — Table S2. The scoring formulation and range of SF-36. (DOCX 12 kb) [file 13561_2019_231_MOESM2_ESM.docx]

**Supplementary Table 2**: The scoring formulation and range of SF-36

| Scale | Formulation | Range |
| --- | --- | --- |
| PF | 3a+3b+3c+3d+3e | 10, 30 |
|  | +3f+3g+3h+3i+3j |  |
| RP | 4a+4b+4c+4d | 4, 8 |
| BP | 7+8 |  |
| GH | 1+11a+11b+11c+11d | 5, 25 |
| VT | 9a+9e+9g+9i | 4, 24 |
| SF | 6+10 | 2,10 |
| RE | 5a+5b+5c | 3, 6 |
| MH | 9b+9c+9d+9f+9h | 5, 30 |

BP: Bodily Pain; GH: General Health; MH: Mental Health; PF: Physical Function; RE: Role emotional; RP: Role Physical; SF: Social Function; VT: Vitality
