# Supplementary material for: Cost-utility analysis of total knee arthroplasty for osteoarthritis in a regional medical center in China
Source: Health Econ Rev. 2019 May 27;9:15. doi: 10.1186/s13561-019-0231-0 (PMC6734290; doi:10.1186/s13561-019-0231-0)
Supplement: Supplementary file 3 — Table S3. SF-36 weights of 8 domain scales. (DOCX 12 kb) [file 13561_2019_231_MOESM3_ESM.docx]

**Supplementary Table 3**: SF-36 weights of 8 domain scales.

| Aspect | Weight |
| --- | --- |
| Physical Function, PF | 0.1001 |
| Role Physical, RP | 0.1131 |
| Bodily Pain, BP | 0.0871 |
| General Health, GH | 0.1341 |
| Vitality, VT | 0.1572 |
| Social Function, SF | 0.0901 |
| Role Emotional, RE | 0.1381 |
| Mental Health, MH | 0.1802 |
